# Emergence of a super-synchronized mobbing state in a large population of coupled chemical oscillators

**DOI:** 10.1038/srep19186

**Published:** 2016-01-12

**Authors:** Gourab Ghoshal, Alberto P. Muñuzuri, Juan Pérez-Mercader

**Affiliations:** 1 Department of Earth and Planetary Sciences. Harvard University, Cambridge, MA 02138, USA; 2 The Santa Fe Institute, 1399 Hyde Park Road, Santa Fe, NM 87501, USA

## Abstract

Oscillatory phenomena are ubiquitous in Nature. The ability of a large population of coupled oscillators to synchronize constitutes an important mechanism to express information and establish communication among members. To understand such phenomena, models and experimental realizations of globally coupled oscillators have proven to be invaluable in settings as varied as chemical, biological and physical systems. A variety of rich dynamical behavior has been uncovered, although usually in the context of a single state of synchronization or lack thereof. Through the experimental and numerical study of a large population of discrete chemical oscillators, here we report on the unexpected discovery of a *new* phenomenon revealing the existence of *dynamically distinct* synchronized states reflecting different degrees of communication. Specifically, we discover a novel large-amplitude super-synchronized state separated from the conventionally reported synchronized and quiescent states through an unusual sharp jump transition when sampling the strong coupling limit. Our results assume significance for further elucidating globally coherent phenomena, such as in neuropathologies, bacterial cell colonies, social systems and semiconductor lasers.

The phenomenon of synchronization among coupled oscillators is fairly ubiquitous in natural and manmade systems. Examples in biology include the synchronized flashing of fireflies^1^, the chirping of crickets^2^, cardiac pacemakers^3^, yeast cells^4^ and the firing of neurons^5^. In social systems coherence occurs in cooperative crowd effects^6^,^7^, while in non-living physical systems synchronization is seen, for example, in arrays of Josephson junctions^8^ and semiconductor lasers^9^. Last, but not least, systems of coupled chemical reactions^10^ provide representative examples in chemistry.

Precise characterizations of synchronization have been made through analytical considerations (phase models based on the Kuramoto-family of models^11^), while coupled electrochemical oscillators^12^, reactors^13^ and well-mixed populations of catalyst-loaded oscillatory beads have proven to be excellent experimental templates^14^. The latter is particularly interesting, being scaleable to a large population and known to have a panoply of dynamical behaviors ranging from phase synchronization^15^ to amplitude-entrainment through external driving^16^ to quorum sensing effects^17^.

Because of their rich phenomenology, systems of globally coupled beads provide an ideal setting to investigate potentially new dynamical behavior. In particular, there are few experimental instances demonstrating non-trivial dynamics *beyond* the synchronization transition, with the notable exception being oscillator death^18^ where an initially synchronized population abruptly ceases oscillations with increased coupling strength. Furthermore, while a large amount of effort has been dedicated to examining transitions to synchronization, relatively little is known about the potential existence of multiple *states* of synchronization. Examples abound in nature, such as the observation that the frequency of synchronized crickets adjusts according to the ambient temperature^2^. In neurology, pathologies are known to occur due to abnormal synchronization in pyramidal neuronal cells, so called Interictal Epileptogenic Discharges^19^. These, however, are distinct states of synchronization from *epileptic seizures* which are global high amplitude patterns found in EEG recordings^20^ occurring when globally connected groups of neurons communicate (through spatial transfer of neurotransmitters) with a *faster* time-scale than that of neural oscillations^21^.

Here we report on our results in the search of multiple synchronized states in a large population of beads loaded with ferroin (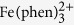) as a catalyst and immersed in a catalyst-free Belousov-Zhabotinsky (BZ) solution^22^. Our experimental setup, described in^16^ and SOM Sec. S1, consists of a continuously stirred tank reactor (CSTR), where beads are immersed in the reaction mixture which is then stirred at different rates to adjust their interconnectivity via transport-facilitation of signaling species. A RedOx cycle occurs through the oxidization of ferroin by reagents in the solution resulting in the production of an autocatalyst activator 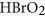 and an inhibitor 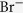. The oxidized ferroin reacts with the solution regenerating the reduced form of the catalyst and the inhibitor, with the cycle repeating itself when the latter falls below a particular threshold. A combination of the stirring rate and bead density represents the coupling strength; if one fixes the latter, then the former plays the role of the control parameter in our system.

## Results

**Figure 1:**
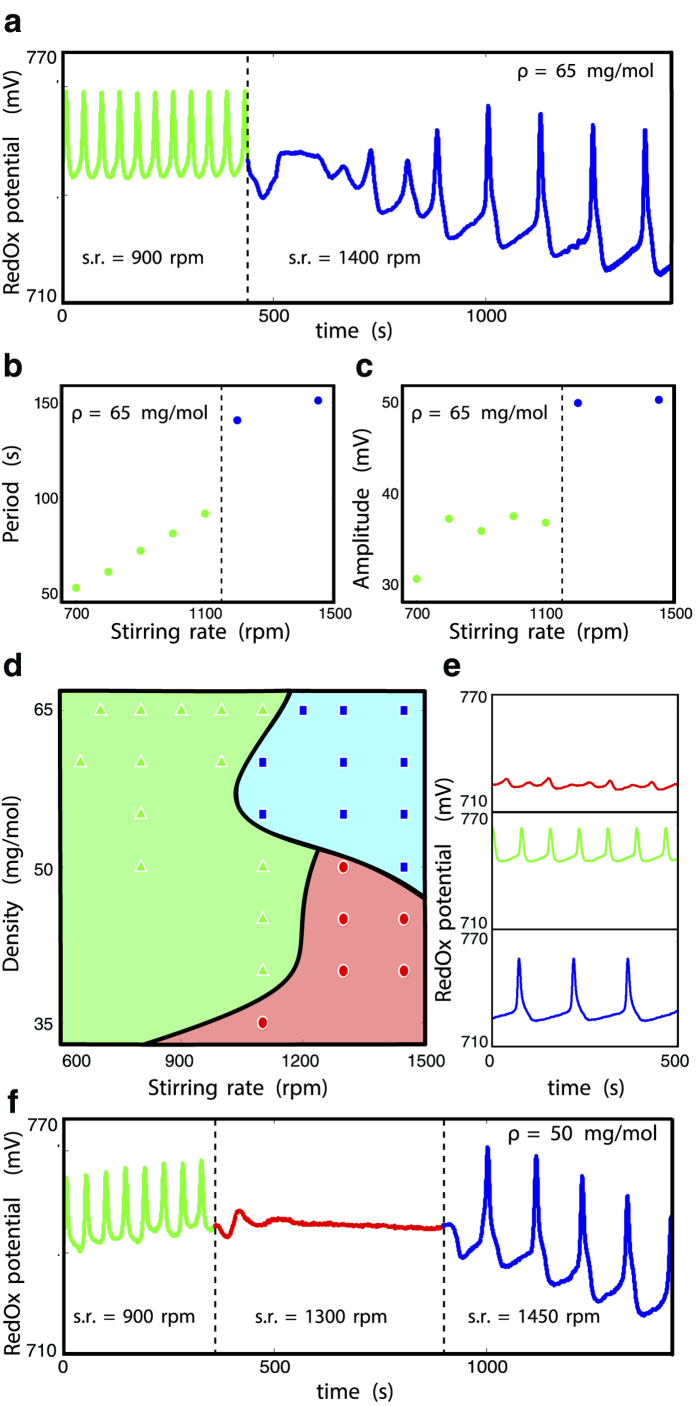
Collective oscillations in our experiment as measured through the RedOx potential. (**a**) The temporal evolution of oscillations with increasing stirring rate (*s.r.*) for a fixed bead density. The vertical dashed line separates two regimes: high frequency, low amplitude oscillations (green) and low frequency, high amplitude oscillations (blue). For the same density, panels (**b**,**c**) show the period of oscillations *T* and amplitude *A* in function of *s*.*r*. (Points represent multiple realizations, error bars smaller than points). Experiments conducted for a wide configuration of the density and stirring rates can be compiled into a putative phase diagram, panel (**d**). The time evolution in each region is shown in (**e**) including a globally quiescent state (red). The phase diagram demonstrates the existence of three distinct dynamical regimes which can be sampled, for example by varying 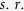 for a fixed density as shown in (**f**) (For variation of period and amplitude as a function of bead-density see Fig. S1).

The resulting collective state was measured through the RedOx potential. In Fig. 1 we plot our experimental results. Panel **a** shows the temporal evolution of the signal with increasing stirring rate in a single experiment with fixed bead density. Two distinct regimes are present: for lower stirring rates (*K* = 900 rpm) one observes low-amplitude high-frequency oscillations (green curve) corresponding to the well-known collective synchronization of the bead oscillations^15^. As one samples the strong coupling regime by increasing the rate (*K* = 1400 rpm) there is a sudden and abrupt emergence of large-amplitude and lower-frequency oscillations (blue curve). This *new regime* is in sharp contrast to the expected dynamics in this regime which was thought to support the existence of a quiescent state (so-called oscillator death)^18^.

In panels **b** and **c** we plot the period and amplitude of oscillations as a function of the stirring rate for the same bead density. Each data point is an average of multiple realizations of the experiment for a given stirring rate up to a maximum of 1500 rpm (which is the limit of our experimental apparatus). Both figures confirm the existence of two distinct synchronization regimes separated by a sharp jump transition in both period (50% increase) and amplitude (25% increase) prompting us to term the blue region as a super-synchronized state or *mobbing* state, similar to an equivalent phenomenon in sociology^7^.

In order to check the robustness of this effect we conducted several experiments for multiple combinations of stirring rate and bead density. The results are compiled in the “phase” diagram shown in Fig. 1d where each point corresponds to multiple realizations of experiments conducted for a fixed pair of density and stirring values. The characteristic time evolution of the RedOx potential in each region shown in Fig. 1e allowed us to demarcate three distinct dynamical behaviors of the system. In addition to the green and blue states one also observes a globally quiescent state (red curve and points) at low bead density and high stirring. Intriguingly the figure suggests that the large amplitude blue state can be accessed directly from the quiescent red state *either* through an increase in density or—for a relatively narrower region of parameter space—an increase in the stirring rate. That is to say, while the strong coupling regime leads to oscillator death (as previously established), still stronger coupling leads to the emergence of the reported mobbing state.

A representative example is shown in Fig. 1f where we plot the time evolution of the RedOx potential for three different stirring rates in the *same* experiment. The three regimes are clearly visible with the green and blue states now separated by an intermediate red state. Indeed, the boundary separating the quiescent and high-frequency oscillatory regimes is reminiscent of a quorum sensing transition as previously reported in^17^. The transition from the red state to the blue state is even more dramatic—the system directly transitions from a steady state to that with large amplitude oscillations—and may be considered a *hyper* quorum sensing effect.

**Figure 2:**
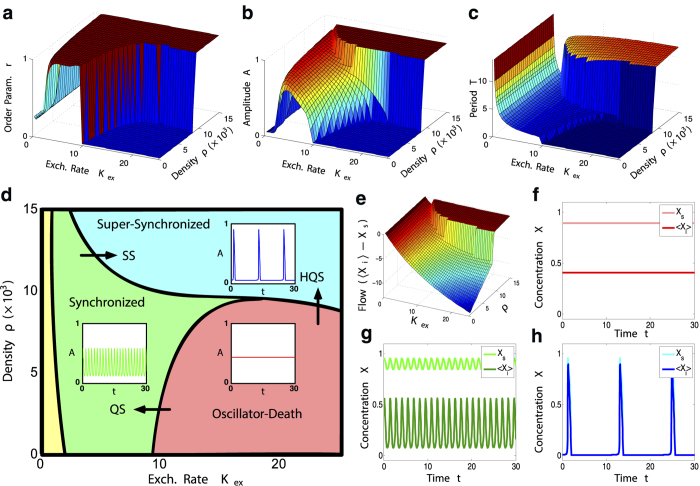
Summary of results from numerical simulations of the oregonator model, Eqns. (1) and (S2), for *n* = 10^3^ beads. Panels (**a**) through (**c**): synchronization order parameter *r* (Eq. (2)), amplitude *A* and period of oscillations *T* for the average concentration of autocatalyst on beads, 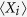, as a function of the exchange rate *K*_*ex*_ and bead-density *ρ*. Collective information from (**a**–**c)** combined into a phase diagram (**d**) marking different regimes of oscillations, color scheme same as in Fig. 1 (yellow marks the region of incoherent oscillations). Insets show characteristic time evolution of 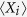 within each regime. Transitions between regimes are marked by arrows and labeled according to three possibilities: Quorum sensing (QS), Hyper-Quorum sensing (HQS) and Super-Synchronization (SS). (**e**) The flow of autocatalyst between the beads and medium in function of 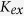 and *ρ*. Panels (**f**) through (**h**) show the temporal evolution of autocatalyst in medium *X*_*s*_ and in beads 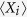 in the different regimes. (For simulation parameter values see SOM Sec. S2).

Insight into the unusual dynamical behavior of the system can be gleaned via the simulation of an idealized numerical model^17^—based on the three-variable oregonator model^23^—that best approximates our experimental setup. Here, for each bead 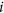, the concentration values for 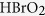 is denoted *X*_*i*_, *Y*_*i*_ for 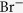 and *Z*_*i*_ for the oxidized catalyst. The *X*_*i*_ evolve according to,


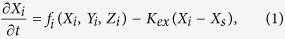


where *f*_*i*_(*X*_*i*_, *Y*_*i*_, *Z*_*i*_) represents the chemical reactions localized on the beads, *K*_*ex*_ accounts for their information exchange rate with the surrounding solution, whose concentration *X*_*s*_ evolves according to Eq. S2 which also includes a density parameter. (For details see SOM Sec. S2). Synchronization in the system can be measured through the absolute value of the complex quantity


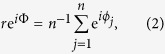


where *ϕ*_*j*_ is the phase of the *j*,th oscillator and Φ that of the synchronized fraction^11^. In Fig. 2 we plot our results. Panel **a** shows the order parameter *r* as a function of density *ρ* and exchange coupling *K*_*ex*_. The figure distinguishes between two regimes, a quiescent state (*r* = 0) and a synchronized state (*r* ≠ 0) but provides *no information* about any *difference* in dynamical behavior within the latter regime. On the other hand, consistent with the experimental observations in Fig. 1b,c, the amplitude *A* and period *T* of oscillations for 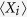, shown in 2**b,c** clearly demarcates the synchronized region into two different dynamical states separated by an abrupt jump transition in both quantities at the same (*ρ*, *K*_*ex*_) boundary.

The combined information in panels **a** through **c** can be displayed as a single phase diagram (Fig. 2d) that shows the different dynamical regions. The experimentally relevant ones are color coded the same as in Fig. 1 with the characteristic time evolution of the concentration shown as insets. Just as in the experiments, three dynamical regimes co-exist: a low amplitude high frequency synchronized state (green) at intermediate exchange rates and densities; a quiescent state (red) exists at high exchange rate but low densities; and finally a large amplitude, low frequency state (blue) at high densities and a wide range of exchange rates.

While the existence of the red and green states has been previously mapped out^17^, the unusual appearance of the blue state can be understood in the context of the flow of autocatalyst between the medium and the beads. In Fig. 2e we plot 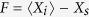 as a function of *ρ* and 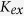. For a wide swathe of parameter space the flow is negative, indicating a greater concentration of 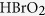 in the medium than in the beads. This difference asymptotically decreases as one traverses phases space from the red to the green state but vanishes abruptly—once again through a jump transition—as the blue state is accessed.

The time evolutions of *X*_*s*_ and 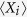 in the red, green and blue regions plotted in Fig. 2f through **h** make this effect more apparent. Both the red and green states are characterized on average by lower concentrations in beads than in the medium, with the main difference being the appearance of oscillations in Fig. 2g. In Fig. 2h, however, the signals for the medium and the bead are practically indistinguishable suggesting an identical phase, period and nearly identical amplitudes.

Thus the primary difference between the green and blue states is the following: in the former case beads synchronize among each other and as the coupling increases eventually reach a state of *full* or *complete* synchronization with each other, sharing a common phase, frequency and amplitude. In this regime, as suggested by Fig. 2g, the dynamics of the medium is distinct from that of the beads immersed in it. However, as the coupling is further increased, in the latter case, there is a *second* dynamical transition where the already *perfectly* synchronized beads now also synchronize with the background medium with a common dynamical signature. To distinguish between the well-known complete synchronization state and the newly observed second dynamical transition reported here, we term the blue state a *super-synchronized* or *mobbing* state reflecting the strong harmony among the fully synchronized beads *with* the medium they are immersed in and whose active role is crucial for reaching this state.

## Discussion

To summarize, we document the existence of a novel dynamical state in a population of coupled discrete chemical oscillators. This super-synchronized or mobbing state—characterized by large amplitude, low frequency oscillations—resides in the strong coupling limit (as measured by information exchange and oscillator density), a particularly surprising result, given the conventional wisdom of a complete cessation of oscillations in this regime. The detailed study of an idealized numerical model suggests the origin of this new mobbing state is a result of the interplay between the dynamics of the beads and the medium in which they are immersed. Specifically, the super-synchronized state is accessible only when the *flow* of signaling species (autocatalyst) between the beads and the medium is minimized, a condition one can achieve either by increasing the density of oscillators, the exchange rate or indeed both.

The dynamical interplay between the oscillators, the medium and the rate of information exchange is reminiscent of global coherence phenomena in neurology such as in epilepsy or mobbing crowds in social networks^7^. Indeed, the abruptness of the super-synchronization transition may have implications in biology where synchronization plays an important role in many contexts, including the cell cycle^24^ and cooperative behavior in bacterial cell colonies^25^. In addition, the system reaches a high level of self-organization as a consequence of the participation of the medium in the process of oscillator synchronization, brought about by the strong coupling between oscillators. Of course, this behavior is analogous to the so-called mobbing behavior found in hyper-communicated social media when the individuals not only have access to their immediate neighbors but they communicate with distant neighbors via social media.

We also note that the degree of information exchange between the oscillators (coupling strength) serves as a “switch” to access the different states and their regimes, suggesting intriguing engineering applications in similar physical systems. Finally our description may serve as a template to explain some natural phenomena such as the observation that the frequency of synchronized chirps in grasshoppers increases with temperature^2^.

## Additional Information

**How to cite this article**: Ghoshal, G. *et al.* Emergence of a super-synchronized mobbing state in a large population of coupled chemical oscillators. *Sci. Rep.* **6**, 19186; doi: 10.1038/srep19186 (2016).

## Supplementary Material

Supplementary Information
