# Supplementary material for: Emergence of a super-synchronized mobbing state in a large population of coupled chemical oscillators
Source: Sci Rep. 2016 Jan 12;6:19186. doi: 10.1038/srep19186 (PMC4709686; doi:10.1038/srep19186)
Supplement: Supplementary Information [file srep19186-s1.pdf]

Supporting online material

Emergence of a super-synchronized mobbing state in a large population of coupled chemical oscillators

Gourab Ghoshal, Alberto P. Muñuzuri and Juan Pérez-Mercader

Table of Contents

S1 Experiments **S-2**
S1.1 Setup and Materials S-2
S1.2 Experimental procedure S-2

S2 The three-variable Oregonator Model **S-2**

S3 Supplementary References **S-5**

List of Figures

S1 Period and Amplitude of oscillations in function of bead density S-3
S2 Initial conditions for simulations of Oregonator model S-4

S1 Experiments

S1.1 Setup and Materials

Experiments were conducted using cation-exchange beads DOWEX-50WX41with radius $\sim 100\ \mu m$. The beads were sourced from SIGMA Cat. No. 428663 and were loaded with Ferroin as a catalyst ($Fe(phen)_3^{2+}$) (using Ferroin indicator solution SIGMA CAT. No 46270-250ML) and following the procedure described in [S1]. The dried, catalyst loaded beads were then dispersed in a reactor containing 20 ml of catalyst-free BZ reaction solution ([$CH_2(COOH)_2$] = 0.135M, [NaBrO3] = 0.41M, [NaBr] = 1.8M, [H2SO4] = 2.6M).

A glass jacketed beaker was employed as the reactor (with the reacting chamber inside the jacketed beaker) and thermostatted water was made to circulate—using a bath VWR MX7LL R-20—through the jacket in order to control temperature. Temperature was maintained at $27.5 \pm 0.3\,°C$ for all configurations of the experiment reported here. The chemicals as well as loaded beads were immersed in the chamber and were magnetically stirred (IKA RCT basic) with a cross-bar (SIGMA Z284084) placed immediately below the beaker.

In addition two sensors were placed within the reactor: a temperature probe from Omega (EI1510101/RTD) and a platinum electrode to record the RedOx potential.

S1.2 Experimental procedure

Experiments were conducted according to the following standard procedure. Once the beads were loaded with the catalyst and dried, they were placed in the reacting chamber together with the rest of the chemicals involved in the BZ reaction. The magnetic cross bar was adjusted to a specific stirring rate and the system was allowed to evolve for a transient time (typically 2 hours) till stable oscillations persisted in the system. Following this, periods and oscillation amplitudes were extracted from the RedOx signal which was continuously measured for 3 hours. In Figure S1 we show the period and amplitude as a function of bead density for two different stirring rates.

S2 The three-variable Oregonator Model

The simulations presented in Figure 2 of the main text were performed using the 3-variable photosensitive Oregonator model that describes the catalyzed Belousov-Zhabtoinsky reaction [S2] operating on each bead i. The evolution equations for this model are,

$$\epsilon \frac{\partial X_i}{\partial t} = X_i (1 - X_i) + Y_i (q - X_i) + p_2 \gamma - K_{ex} (X_i - X_s), \tag{S1a}$$

$$\epsilon' \frac{\partial Y_i}{\partial t} = 2hZ_i - y_i (q - X_i) + p_1 \gamma - K_{ex} (Y_i - Y_s), \tag{S1b}$$

$$\frac{\partial Z_i}{\partial t} = X_i - Z_i + \left(\frac{p_1}{2} + p_2\right) \gamma, \tag{S1c}$$

where X_i, Y_i and X_i are dimensionless variables representing the concentrations of $HBrO_2$, Br^- and the catalyst. There are n beads in the system and the projected light intensity is characterized by a proportionality constant γ. Finally, q, h, ϵ, ϵ', p_1 and p_2 are parameters related to reaction rates

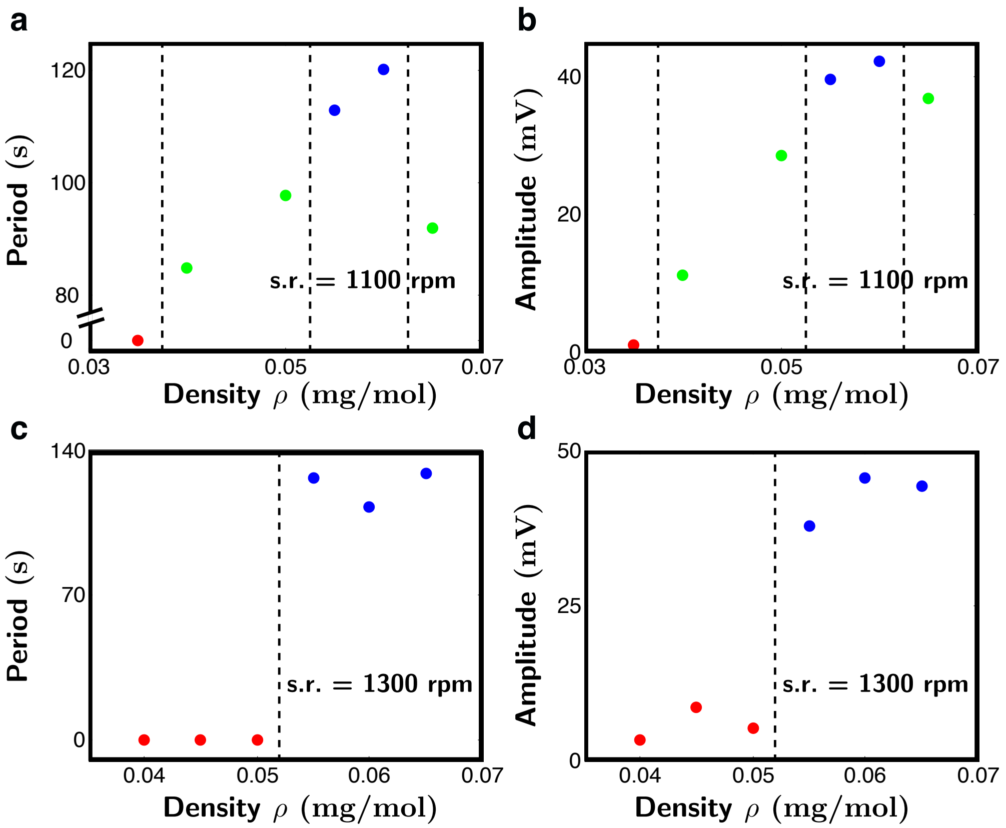

Figure S1: **(a)** Period of oscillations as a function of bead density for a fixed stirring rate. Color scheme same as in main manuscript. (b) Amplitude for the same system. (c) and (d) same as in (a) and (b) but now for a higher stirring rate.

and initial concentrations. The parameter values for each bead were adjusted differently to account for the fact that non-uniform bead sizes in experimental conditions confers different oscillation frequencies ω_i for each bead i.

The effect of a discrete population of active beads embedded in a non-catalyzed BZ solution was taken into account according to the procedure outlined in [14] where one introduces the variables X_s and Y_s corresponding to the concentrations of the X_i and Y_i in the surrounding solution (Z_s does not appear, as the catalyst is immobilized at the beads and cannot diffuse into the surrounding solution). These evolve according to

$$\epsilon \frac{\partial X_s}{\partial t} = X_s (1 - X_s) + Y_s (q - X_s) + \frac{\langle V \rangle_n}{V_s} K_{ex} \sum_i (X_i - X_s)$$
$$\epsilon' \frac{\partial Y_s}{\partial t} = -Y_s (q - X_s) + \frac{\langle V \rangle_n}{V_s} K_{ex} \sum_i (Y_i - Y_s) , \qquad \text{(S2)}$$

where K_{ex} is the exchange rate between the beads and the surrounding solution, $\langle V \rangle_n$ is the average volume of a bead, V_s is the volume of the solution, with the ratio of the two acting as a dilution

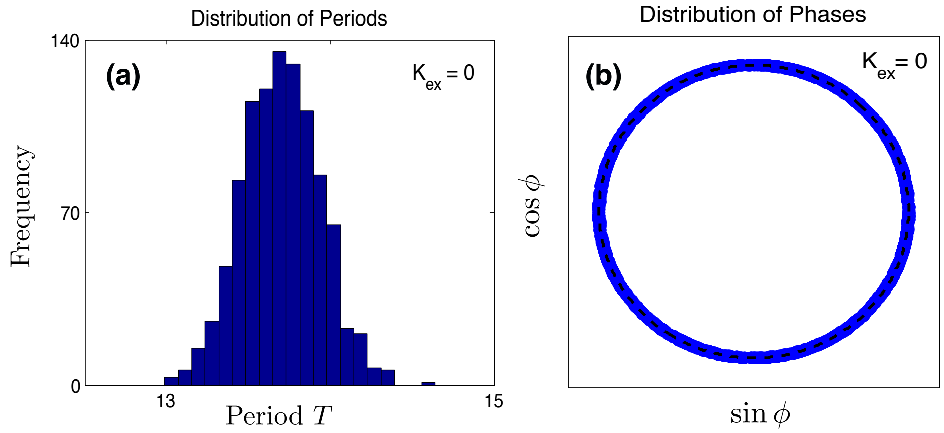

Figure S2: **(a)** Distribution of oscillation periods T for $n = 10^3$ beads where $K_{ex} = 0$. The distribution was generated as a consequence of the following parameter values: $\epsilon = 0.01 \pm 0.0$, $\epsilon' = 0.015 \pm 0.0$, $q = 0.002 \pm 0.0$, $h = 0.70 \pm 0.03$, $p_1 = 1$, $p_2 = 1$, $\gamma = 0$, $\langle V \rangle_n / V_s = 10^{-5}$. The resultant mean period is $\langle T \rangle = 13.7 \pm 0.2$. **(b)** Distribution of initial phases for the same set of beads chosen from the uniform distribution $P(\phi) \sim 1/2\pi$.

factor. Note the absence of the effect of light intensity γ.

The following parameter values were used for the simulation presented in Figure 2 of the main text: $n = 10^3$, $\epsilon = 0.01$, $\epsilon' = 0.015$, $q = 0.002$, $h = 0.70 \pm 0.03$, $p_{1,2} = 1$, $\gamma = 0$, $\langle V \rangle_n = 10^{-4}$ and $V_s = 10^{-1}$. The differential equations are stiff and were integrated using a fourth order Runge-Kutta scheme.

With these parameter values, the initial distribution of periods for the uncoupled case ($K_{ex} = 0$) is shown in Fig. S2a, while the initial phases are chosen from the uniform distribution as shown in Fig. S2b. The results of the simulations are shown in Figure 2 and the results discussed in the main text.

S3 Supplementary References

S1 K. Miyakawa, H. Isikawa, Phys. Rev. E 2002, **65**, 056206

S2 T. Amemiya, T. Ohmori, M. Nakaiwa, T. Yamaguchi, Journal of Physical Chemistry A **102**, 4537 (1998)
